# Necrotizing Fasciitis in the Kimberley: A Five-Year Retrospective Study From a Remote Australian Health Service

**DOI:** 10.7759/cureus.101425

**Published:** 2026-01-13

**Authors:** Jessica L O'Sullivan, Allison J Duchow

**Affiliations:** 1 General Surgery, East Metropolitan Health Service, Perth, AUS; 2 General Surgery, Western Australia Country Health Service, Perth, AUS

**Keywords:** emergency surgery, general surgery, necrotizing fasciitis, rural and remote health, soft tissue infection

## Abstract

Background

Necrotizing fasciitis (NF) is a rapidly progressive, life-threatening infection with significant morbidity and mortality. Rural and remote regions face unique challenges in the timely diagnosis and management of NF.

Methodology

This retrospective study included all patients diagnosed with NF in the Kimberley region, Western Australia, between June 2020 and June 2025. Data were extracted from medical and surgical records, microbiology databases, and theater logs. Demographic, clinical, microbiological, and outcome variables were analyzed descriptively.

Results

In total, 21 patients were identified during the five-year period. The estimated incidence rate was 12 per 100,000 population. The median age was 49 years (interquartile range = 37-56); 8 (38%) were males, and 13 (62%) were females. Overall, 16 (76%) identified as Aboriginal and/or Torres Strait Islander. Diabetes (76%, 16), obesity (86%, 18), and smoking (62%, 13) were common. The most frequent site of infection was the lower limb, accounting for 52% (11) of cases, followed by the gluteal or buttock in 24% (5). All patients underwent operative management, and 9% (2) of patients died in the hospital.

Conclusions

The incidence of NF in the Kimberley is higher than the national and international average. This presents unique diagnostic and management challenges due to geographic remoteness, workforce shortages, and comorbidity burden. This study contributes valuable insights into the regional epidemiology and the management of NF in rural settings.

## Introduction

Necrotizing fasciitis (NF) is a rare and often fatal soft tissue infection characterized by severe infection of the skin and underlying tissues with rapid disease progression. NF is a purulent infection that primarily involves the subcutaneous tissue, superficial fascia, and deep fascia while sparing the superficial muscle layer. The spread leads to thrombus formation within the vasculature, resulting in ischemia, necrosis, and tissue death. The decomposition of the bacteria and their metabolic products gives the disease its characteristic malodor [1]. NF usually presents acutely and progresses rapidly, often accompanied by sepsis, shock, multiorgan dysfunction, and a high risk of death [1,2].

The global estimated incidence of NF is 0.3-15.5 cases per 100,000 population [3]. Australian data estimates an incidence of 1-9 per 100,000; however, this remains heterogeneous due to limited regional reporting [4,5]. A 15-year study in South Australia by Tam et al. reported necrotizing soft tissue infections in 19 of every 100,000 hospital admissions [6]. Disease-related morbidity and mortality are high, with a mortality in remote Australian settings as high as 16-28% [7]. Established risk factors include obesity, smoking, diabetes mellitus, cardiovascular disease, peripheral arterial disease, chronic alcohol use, and intravenous drug use, although almost a quarter of patients have no significant underlying comorbidities [1,3-5].

Causative organisms are typically classified as type I (polymicrobial) or type II (monomicrobial) infections involving both aerobic and anaerobic bacteria [6]. The most commonly offending organisms are *Streptococcus pyogenes* or *Staphylococcus aureus*, although *Clostridium* and *Aeromonas* species are increasingly recognized. NF remains primarily a clinical diagnosis, and prompt recognition of the disease is critical. Diagnosis can be challenging as early features can mimic other soft tissue infections such as cellulitis, erysipelas, or deep vein thrombosis, which can delay treatment and appropriate management [3,8,9]. The mainstay of treatment is urgent surgical debridement combined with broad-spectrum antimicrobial therapy, aggressive fluid resuscitation, and meticulous haemodynamic monitoring [2,9,10]. Multidisciplinary team management is essential in the delivery of appropriate and lifesaving care.

The Kimberley region of Western Australia spans more than 400,000 km² and is among the most remote and socioeconomically disadvantaged regions in the country. The Kimberley includes six major townships. The three largest towns are Broome, Derby, and Kununurra. In 2021, the region’s population was 35,092 compared with 2.66 million for the state, and approximately half of Kimberley residents identify as Aboriginal or Torres Strait Islander [11]. Broome Hospital functions as a secondary-level referral center for much of the region, providing surgical and specialist support for the surrounding communities.

The Kimberley’s vast geography, tropical climate, and limited access to specialist and critical care services pose distinct challenges to the timely diagnosis and management of NF. Despite these factors, no prior data describe the epidemiology, presentation, or outcomes of NF in this region. Most existing Australian data originates from Far North Queensland [7,12]. Hence, this study aims to identify the incidence, risk factors, microbiology, and outcomes of patients with a diagnosis of NF in the Kimberley over a five-year period, highlighting the impact of remoteness and comorbidity burden on disease presentation and management.

## Materials and methods

Patient consent was waived due to the retrospective, observational nature of this research. This study was conducted in conjunction with the region’s Health Service Quality Improvement team. This study was conducted in accordance with the Strengthening the Reporting of Observational Studies in Epidemiology (STROBE) guidelines [13].

In this retrospective study, data were collected from all patients from June 2020 to June 2025 with an International Classification of Diseases, 10th Revision coding diagnosis of “necrotizing fasciitis,” “Fournier’s Gangrene,” or “necrotizing soft tissue infection.”

Data were gathered from both paper and electronic records, review of operative logs, and pathology and microbiology records. Patients were screened for suitability. Patients who were managed within the study period with either histological or operative reports confirming a diagnosis of NF were included in the study. Duplicate data were excluded. Data were entered into an anonymized Excel database. Data were gathered on patient demographics (age, sex, Indigenous status), comorbidity profile (diabetes, obesity, smoking), site of infection, laboratory values (white cell count (WCC), C-reactive protein CRP, lactate), microbiology (wound culture results), management (operative data, transfer to tertiary centre, intensive care unit (ICU)/high-dependency unit (HDU) admission, length of stay in hospital, and mortality. Missing data were not included.

Descriptive statistics were calculated for all variables. Normally distributed continuous variables were summarized as mean ± standard deviation (SD). Non-normally distributed data were presented as median (interquartile range (IQR)). Categorical variables were summarized as frequencies and percentages (%).

Univariable analysis was performed to examine associations between predictor variables for in-hospital mortality. Continuous variables were analyzed using an independent samples t-test to compare means between survivors and non-survivors. Test statistics (t-values) and p-values were reported. For categorical variables, Fisher’s exact test was used between mortality groups. P-values were reported for each comparison.

Univariable logistic regression models were used to estimate associations with in-hospital mortality. Odds ratios (ORs) with 95% confidence intervals (CIs) were calculated for categorical predictors using logistic regression or exact methods. Wald chi-squared statistics were reported for logistic regression models. For continuous variables, mean differences and their 95% CIs were calculated. For variables with perfect separation, Firth's penalized likelihood method was employed where feasible.

Data were analyzed using SPSS version 28.0 software (IBM Corp., Armonk, NY, USA). Graphs and tables were formatted using artificial intelligence software. Data was stored in accordance with WACHS data storage and retention policy.

## Results

Between June 1, 2020, and June 1, 2025, we identified 21 patients with a diagnosis of NF. The median age was 49 years (IQR = 37-56); 8 (38%) were males, and 13 (68%) were females. Overall, 16 (76%) identified as Aboriginal and/or Torres Strait Islander. Diabetes mellitus was present in 16 (76.2 %), obesity in 18 (85.7%), and 13 (61.9%) were active smokers. The site of infection was categorized as upper limb, lower limb, perineum (Fournier’s gangrene), buttock/perianal region, and trunk. The most common anatomical site was the lower limb, accounting for 52% (11) of cases, followed by the gluteal or buttock at 24% (5). Fournier’s gangrene and abdominal wall infections occurred in 10% (2) of cases, while upper limb involvement occurred in only 5% (1) of cases. A summary of baseline characteristics is illustrated in Table 1.

**Table 1:** Baseline characteristics, comorbidities, site of infection, and outcomes of patients with necrotizing fasciitis in the Kimberley (n = 21). All values are expressed as n (%) unless otherwise specified. IQR = interquartile range; ICU = intensive care unit; HDU = high-dependency unit

Variable	n (%) or median (IQR)
Demographics	
Age, years	49 (37–56)
Male sex	8 (38%)
Aboriginal and/or Torres Strait Islander	16 (76%)
Comorbidities	
Diabetes mellitus	16 (76%)
Obesity	18 (86%)
Current smoker	13 (62%)
Site of infection	
Lower limb	11 (52%)
Gluteal/Buttock	5 (24%)
Fournier’s gangrene (perineum)	2 (10%)
Abdomen/Trunk	2 (10%)
Upper limb	1 (5%)
Operative and clinical outcomes	
Operated within 24 hours	8 (38%)
Required tertiary transfer	15 (71%)
ICU/HDU admission	7 (33%)
Amputation	3 (14%)
Length of stay, days	19 (15–28)
In-hospital mortality	2 (9%)

The median WCC at presentation was 20 × 10⁹/L, and the median CRP was 271 mg/L. Laboratory results are summarized in Table 2.

**Table 2:** Summary statistics: WCC, CRP, and lactate. Values are reported as mean and median after exclusion of missing data. WCC = white cell count; CRP = C-reactive protein

Parameter	Mean ± SD	Median (IQR)	Reference range
WCC (×10⁹/L)	20.62 ± 5.59	20.00 (16.00–22.00)	4.0–11.0
CRP (mg/L)	262.86 ± 99.10	271.00 (200.00–311.00)	<5
Serum lactate (mmol/L)	1.61 ± 0.77	1.70 (1.00–2.05)	0.5–2.0

Operative management was performed on all patients. Eight (38%) patients underwent their first operative debridement within 24 hours of presentation. Overall, 71% (15) required transfer to a tertiary center, and 33% (7) required ICU/HDU-level care. Amputation was performed in 14% (3) of cases. The median length of stay in the hospital was 19 days (IQR = 15-28 days).

Wound cultures were positive in all cases. The most isolated organisms were *Staphylococcus aureus* (33%, including 3 cases of methicillin-resistant *Staphylococcus aureus* (MRSA)) and *Streptococcus* species (29%). Polymicrobial infections were frequent, with mixed aerobic and anaerobic organisms identified in 24% (5) of samples. Less frequently isolated organisms included *Pseudomonas aeruginosa*, *Klebsiella pneumoniae*, *Proteus*, and *Escherichia coli*. These findings are summarized in Table 3.

**Table 3:** Wound culture results from patients with necrotizing fasciitis (n = 21). MRSA = methicillin-resistant *Staphylococcus aureus*; MSSA = methicillin-sensitive *Staphylococcus aureus*

Organism/Group	Frequency (n)	Percentage (%)	Notes
*Staphylococcus aureus* (including MRSA, MSSA)	7	33	Includes 3 MRSA-positive cases
*Streptococcus* species (Group A, G, anginosus)	6	29	Often co-isolated with *Staphylococcus* spp.
Pseudomonas aeruginosa	2	10	One meropenem-resistant isolate
*Proteus* species	1	5	—
Klebsiella pneumoniae	1	5	—
Escherichia coli	1	5	Mixed infection
Corynebacterium striatum	1	5	Mixed infection
*Lactobacillus*/*Pichia kudriavzevii*	1	5	Mixed infection
Mixed anaerobes (unspecified)	5	24	Often co-isolated with aerobes
Total	21	100	—

In-hospital mortality occurred in 2 (9%) patients. Table 4 presents the comparison of baseline characteristics and clinical variables between survivors and non-survivors. In this small cohort, the strongest associations were seen with mortality, amputation (OR = 19.00), elevated inflammatory markers (mean CRP = 407 vs. 252 mg/L), and elevated WCC (mean = 27.5 vs. 19.7 × 10⁹/L). Both deaths occurred in patients with diabetes who required amputation.

**Table 4:** Comparison of survivors versus non-survivors. ^†^: Independent samples t-test; ^‡^: Fisher’s exact test; ^§^: statistical testing not performed due to only one observation in the death group WCC = white cell count; CRP = C-reactive protein; ICU = intensive care unit; HDU = high-dependency unit; SD = standard deviation

Variable	Survived (n = 19)	Died (n = 2)	Test statistic	P-value*
Demographics				
Age (years), mean ± SD	47.8 ± 10.5	42.5 ± 10.6	t = 0.40^†^	0.694
Male sex, n (%)	6 (31.6)	1 (50.0)	—	0.598^‡^
Comorbidities				
Diabetes mellitus, n (%)	15 (78.9)	2 (100)	—	1.000^‡^
Obesity, n (%)	16 (84.2)	2 (100)	—	1.000^‡^
Laboratory markers				
WCC (×10⁹/L), mean ± SD	19.7 ± 5.7	27.5 ± 7.8	t = 1.35^†^	0.196
CRP (mg/L), mean ± SD	252.3 ± 104.1	407.0 ± 124.5	t = 1.34^†^	0.197
Lactate (mmol/L), mean ± SD^§^	13.5 ± 9.6 (n = 11)	21.5 (n = 1)	—	—
Disease characteristics				
Lower limb location, n (%)	9 (47.4)	1 (50.0)	—	1.000^‡^
Perineum/Groin location, n (%)	7 (36.8)	0 (0)	—	0.524^‡^
Trunk location, n (%)	4 (21.1)	1 (50.0)	—	0.381^‡^
Management factors				
Surgery <24 hours, n (%)	7 (36.8)	1 (50.0)	—	1.000^‡^
Second operation, n (%)	16 (84.2)	2 (100)	—	1.000^‡^
Amputation, n (%)	2 (10.5)	2 (100)	—	0.056^‡^
ICU/HDU admission, n (%)	6 (31.6)	1 (50.0)	—	0.598^‡^

Table 5 presents the results of univariable logistic regression analysis for predictors of in-hospital mortality. The small number of mortality events (n = 2) limits formal statistical testing and calculation of reliable CIs (p < 0.05).

**Table 5:** Univariable analysis of predictors of in-hospital mortality. ^†^: Odds ratio infinite due to perfect separation (zero events in unexposed group); Wald statistic not calculable; ^‡^: calculated using Firth’s penalized likelihood method due to quasi-complete separation. CI = confidence interval; ICU = intensive care unit; HDU = high-dependency unit

Predictor	Events/Total exposed	Events/Total unexposed	Odds ratio	95% CI	Wald χ²	P-value*
Demographics						
Male sex	1/7 (14.3%)	1/14 (7.1%)	2.17	0.12-38.5	0.28	0.598
Comorbidities						
Diabetes mellitus	2/17 (11.8%)	0/4 (0%)	∞^†^	—	—	1.000
Disease characteristics						
Lower limb location	1/10 (10.0%)	1/11 (9.1%)	1.11	0.06-21.0	0.01	0.945
Trunk location	1/5 (20.0%)	1/16 (6.3%)	3.75	0.20-69.6	0.77	0.381
Management factors						
Surgery <24 hours	1/8 (12.5%)	1/13 (7.7%)	1.71	0.09-32.0	0.14	0.711
Second operation	2/18 (11.1%)	0/3 (0%)	∞^†^	—	—	1.000
Amputation	2/4 (50.0%)	0/17 (0%)	19.00^‡^	0.90-400	3.64	0.056
ICU/HDU admission	1/7 (14.3%)	1/14 (7.1%)	2.17	0.12-38.5	0.28	0.598

## Discussion

In this study, we identified a cohort with a significantly higher than average burden of necrotizing soft tissue infections in the Kimberley region of Western Australia. The estimated incidence of approximately 12 per 100,000 individuals is notably greater than rates reported in other urban and rural Australian and international settings (1-9 per 100,000) [5,7,9,14]. The majority of patients in our study identified as Aboriginal and/or Torres Strait Islander (76%), compared with approximately half of the patients in comparable Australian studies by Bailey et al. and Whitehouse et aI. [5,12]. This reflects the demographic composition of the Kimberley, which has a large First Nations population and a high prevalence of chronic disease.

The anatomical site of infection in the study mirrors published data with the lower limb most frequently affected (52% vs. 57-73%), followed by the gluteal and perineal regions (34% vs. 13-40%) and less frequently the trunk (10% vs. 13-40%), upper limb, and head and neck [3]. This pattern likely reflects regional risk factors, including diabetes, smoking, obesity, and delayed presentation due to geographic remoteness.

The Eastern Association for Surgery of Trauma currently recommends surgical debridement within 12 hours of presentation [15]. In our cohort, just over a third (38%) of our patients underwent operative debridement within the first 24 hours of presentation. This delay is likely multifactorial, reflecting a combination of diagnostic uncertainty, non-specific early symptoms, and limited diagnostic or surgical resources. These findings are consistent with other Australian studies where interhospital transfer is frequently required before definitive surgical management [16]. Diagnostic challenges are compounded by the fact that fewer than half of the patients present with the classical triad of pain out of proportion, swelling, and crepitus, furthering the diagnostic dilemma and leading to potentially fatal delays in recognition of such a rapidly progressing disease [3].

Diagnostic limitations are further magnified in remote regions such as the Kimberley. Many patients first present to small health clinics or district hospitals with limited access to urgent laboratory testing, imaging, or specialist surgical staff. Transport to larger regional or tertiary hospitals depends on the services and availability of the Royal Flying Doctors service, who face logistical constraints related to weather, aircraft availability, competing emergencies, and distance. These realities underscore the importance of clinical acumen and early surgical involvement when NF is suspected.

Various scoring systems have been proposed to aid in the prompt diagnosis of NF, and their effectiveness has been demonstrated in some clinical settings. The Laboratory Risk Indicator for Necrotizing Fasciitis (LRINEC score), developed by Wong et al., showed good predictive value (92% positive, 96% negative) in the original Singaporean cohort; however, its performance has been inconsistent in Australian populations [8,14,17]. Sacks et al. found significantly lower predictive accuracy, limiting its validity for local practice [17]. Furthermore, its reliance on laboratory parameters limits its utility in resource-constrained environments. The Necrotizing Soft Tissue Infections (NECROSIS) score, although developed for surgical triage, lacks practicality in regions with limited surgeon availability [8].

Imaging modalities, including ultrasound, CT, and MRI, can aid in the diagnosis of NF. Ultrasound, as a bedside diagnostic tool, can help rule out other pathologies such as deep vein thrombosis, but it is limited by operator-dependent variability. CT has a reported sensitivity up to 89% and specificity of 93%, with the pathologic signs of gas formation, inflammation, and tissue edema often noted. MRI remains the gold standard imaging modality for diagnosing NF [18]. Both are scarce in the Kimberley, where only two CT scanners are available in the whole region, and no MRI services currently exist. Importantly, the guidelines do not firmly recommend imaging to confirm a diagnosis and disregard their use if it delays time to surgery and lifesaving treatment [10].

Early and aggressive surgical debridement remains the cornerstone of NF management. Current guidelines advocate for surgery within six hours of diagnosis, and re-exploration within 12-24 hours after the initial debridement. Early repeat debridement has been shown to significantly lower the mortality rate from 33% to 7% [19].

In this study, delays to surgery were largely logistical rather than clinical. The secondary referral hospital for the region provides limited capability, non-invasive ventilation and vasopressor therapy, but lacks facilities for mechanical ventilation, dialysis, or specialist intensive care, often necessitating transfer to tertiary centers.

However, the decision to transfer must be carefully considered, balancing clinical stability, surgical expertise, and resource availability. Literature suggests that patients transferred before initial debridement have almost double the mortality rates and significantly worse outcomes compared to those treated initially at the presenting hospital [20]. Conversely, those debrided locally and then transferred show improved outcomes due to better access to multidisciplinary care [9,21]. A major Sydney tertiary center review (2001-2010) found that 38-40% of patients were not debrided before transfer, reflecting a national challenge in timely NF management for rural and remote patients [9].

Postoperative morbidity (multi-organ failure, shock, secondary infection, amputation, etc.) remains high, with some Australian series reporting complications in almost 50% of patients [14]. In contrast, our study showed lower ICU admission and mortality rates than previously reported in the literature [6,2]. A large-scale study conducted in Sydney from 2001 to 2011 reported ICU admission rates at almost 70% [14]. This may be attributed to the improvements in earlier antimicrobial initiation, ward-based care, and evolving treatment protocols [16]. The mortality rate in our study (9%) was lower than that of a comparable population in Far North Queensland (14%) and metropolitan centers (16-25%) [12,16]. Amputation rates (14%) were lower than those reported internationally (25%) and in other Australian studies (10-23%) [3,12]. Notably, previous research has not shown significant differences in mortality rates between Aboriginal and Torres Strait Islander versus non-Aboriginal and Torres Strait Islander groups [12]. Interestingly, studies have shown that the use of vacuum-assisted closure dressing postoperatively significantly lowers mortality rates; however, it has no effect on postoperative morbidity [22].

Broad-spectrum antimicrobial therapy should be initiated promptly when suspecting a diagnosis of NF. In our study, the predominant empirical regime was piperacillin-tazobactam (Tazocin) plus vancomycin, aligning with national guidelines and high local MRSA prevalence. Alternative regimens are recommended in the case of penicillin allergy, the need for renal dose adjustment, or other contraindications [23].

Polymicrobial infections were common (24%), consistent with type I NF patterns reported in other Australian regions [3,6,7]. Monomicrobial infections accounted for only 10% of necrotizing soft tissue infections in our study, lower than that reported in the literature (19-36%). *Staphylococcus* *aureus* and MRSA were the most frequently isolated bacteria, in keeping with the high prevalence of MRSA locally. Studies have shown no correlation between the site of infection and the bacteria involved [14]. Data have shown a lower mortality rate associated with polymicrobial (type 1) infection compared to monomicrobial (type 2) infection, likely due in part to the virulence factors associated with *Streptococcus pyogenes* and *Staphylococcus* *aureus*, which are the most commonly isolated monomicrobial organisms [6].

Early prescription of broad-spectrum antibiotics has been shown in Australian studies to lower the mortality rate from 35-25% to 10% when combined with early surgical intervention, emphasizing the importance of empirical antibiotics in high-risk rural settings [6]. Adjunct treatments such as hyperbaric oxygen therapy and intravenous immunoglobulin remain contentious and are largely unavailable in rural communities due to cost and infrastructure limitations [24].

Our findings highlight a significant comorbidity burden among affected patients, diabetes (76%), obesity (86%), and smoking (62%), all exceeding rates reported in other Australian NF studies, where diabetes ranges from 34-56%, obesity around 26%, and smoking 23-36% [5,12]. These risk factors are well-recognized contributors to disease susceptibility and poorer outcomes, with a higher American Society of Anesthesiologists grade being linked to an increased risk of mortality [25]. Regional health data from WACHS confirm a higher background prevalence of smoking (21%) and diabetes (10%) in the Kimberley compared with national averages (11% and 5%, respectively) [11]. Additionally, socioeconomic disadvantage and food insecurity contribute to high obesity rates, with fresh produce costing up to 30-40% more than in metropolitan Perth [11].

Surgical workforce shortages in rural and remote regions represent a major barrier to the timely management of NF. The Australian Bureau of Statistics data states an average of 7.82 surgeons per 100,000 individuals, compared with 5.97 per 100,000 in Western Australia. Concerningly, the Kimberley, an MM7 (most remote) region, has only 0.07 general surgeons per 100,000 individuals, representing a profound inequity [11].

A study by Stewart et al., who analyzed the data of patients who had died from NF over a nine-year period, found that delays in diagnosis (68%) and surgery (17%) were the leading contributors to NF mortality in Australia [21]. Factors underpinning these delays include limited clinician experience with NF, high staff turnover, inadequate training for non-specialist clinicians, and resource limitations such as equipment, bed pressures, theater staff, and retrieval availability [21,26].

Taken together, these findings emphasize the compounded impact of delayed diagnosis, limited surgical access, and high comorbidity burden on outcomes of NF in remote Australia. To address these challenges, the development of regional protocols for early recognition, triage, and retrieval is essential. Improved coordination will be critical to reducing operative delay and improving survival for patients with NF in the Kimberley and similar rural contexts.

Limitations

Limitations of this study include its retrospective design, small sample size, and potential bias from un-captured confounders. The small sample size limited the power to detect statistically significant associations. Time to surgery was not captured in this study due to the heterogeneous documentation of operation reports and theater logs.

## Conclusions

NF in remote communities presents unique diagnostic and management challenges due to geographic remoteness, workforce shortages, and comorbidity burden. Despite these hurdles, outcomes can be improved through heightened clinical vigilance, early empiric antimicrobial therapy, timely surgical intervention, and interhospital collaboration. It is vital for all frontline clinicians, including general practitioners, junior doctors, nurses, physicians, and surgeons from all specialities, to have NF at the forefront of their minds when diagnosing soft tissue pathologies. This study contributes valuable insights into the regional epidemiology and the management of NF in rural and remote settings. It highlights the ongoing need for health equality, education, and infrastructure investment in rural and remote communities.
